# Circulating sRANKL, Periostin, and Osteopontin as Biomarkers for the Assessment of Activated Osteoclastogenesis in Myeloma Related Bone Disease

**DOI:** 10.3390/cancers15235562

**Published:** 2023-11-24

**Authors:** Vladimir Gerov, Daniela Gerova, Ilina Micheva, Miglena Nikolova, Milena Pasheva, Neshe Nazifova, Bistra Galunska

**Affiliations:** 1 Clinics of Hematology, “St. Marina” University Hospital, 9010 Varna, Bulgaria; vladimirgerov@gmail.com (V.G.); ilinamicheva@gmail.com (I.M.); 2 Department of Clinical Laboratory, Faculty of Medicine, MU Varna, 9002 Varna, Bulgaria; 3 Second Department of Internal Disease, Faculty of Medicine, MU Varna, 9002 Varna, Bulgaria; 4 Department of Biochemistry, Molecular Medicine and Nutrigenomics, Faculty of Pharmacy, MU Varna, 9000 Varna, Bulgaria; miglena.todorova@mu-varna.bg (M.N.); milena.pasheva@mu-varna.bg (M.P.); neshe.ferahova@gmail.com (N.N.); bistra.galunska@gmail.com (B.G.)

**Keywords:** sRANKL, periostin, osteopontin, myeloma bone disease, biomarkers

## Abstract

**Simple Summary:**

Multiple myeloma (MM) accounts for about 10% of all hematologic malignancies and is usually accompanied by severe bone disease. We analyzed the circulating levels of sRANKL, periostin, and osteopontin as osteoclast activators in newly diagnosed MM (NDMM) patients at diagnosis and in the course of treatment. Given the significantly higher levels of tested proteins at diagnosis, their correlations with the stage and grade of the disease, as well as their gradual decrease in the course of treatment, we suggest the applicability of these proteins as potential biomarkers for myeloma related bone disease (MBD) monitoring.

**Abstract:**

The hallmark of multiple myeloma is myeloma related bone disease. Interactions between myeloma plasma cells (MPCs), stromal cells, and the bone marrow (BM) microenvironment play a critical role in the pathogenesis of MBD. Bone remodeling is severely dysregulated with the prevalence of osteoclast activity. We aimed to assess circulating levels of sRANKL, periostin, and osteopontin as osteoclast activators in NDMM patients at diagnosis and in the course of treatment, correlations with clinical and laboratory data, and to evaluate their potential as additional biomarkers for the assessment of MBD. The current study involved 74 subjects (41 NDMM patients, 33 controls). MBD was assessed by whole-body low-dose computed tomography. sRANKL, periostin, and osteopontin were assayed by commercial ELISA kits. At diagnosis, all tested parameters were significantly higher in NDMM patients compared to the controls (*p* < 0.0001), correlating with disease stage, MBD grade, and BM infiltration by MPCs. During therapy, the serum levels of all tested proteins decrease, most prominently after autologous stem cell transplantation (*p* < 0.0001). A significant reduction was established in patients achieving complete and very-good partial response compared to all others (*p* < 0.05). In conclusion, sRANKL, periostin, and osteopontin reflect MBD severity and could be promising markers for MBD monitoring and the effect of myeloma treatment.

## 1. Introduction

Multiple myeloma (MM) is a plasma cell disorder with increasing incidence worldwide in recent decades [1]. It accounts for about 10% of all hematologic malignancies [2]. The uncontrolled proliferation of abnormal monoclonal plasma cells (MPCs) in bone marrow (BM) impels the development of myeloma related bone disease (MBD). At diagnosis, up to 80% of patients present with osteolytic bone lesions [3] and are at increased risk of skeletal-related events (SRE) such as pathological fractures and spinal cord compression. Bone complications lead to a significant reduction of quality of life and place a substantial additional burden on patients and healthcare systems [4]. Complex interactions between MPCs, stromal cells, and the BM microenvironment play a critical role in the pathogenesis of MBD [5,6]. Many signaling pathways including Notch signaling, Wnt/β-Catenin signaling, RANKL/RANK/OPG (receptor activator of nuclear factor kappa-Β ligand/receptor activator of NF-κB, osteoprotegerin) axis are activated [7,8]. Bone remodeling is severely dysregulated, manifested by increased osteoclast activity and suppressed osteoblast activity resulting in osteolytic lesions and bone destruction [9].

RANKL/RANK signaling is vital for osteoclastogenesis. As a membrane protein, RANKL is highly expressed in osteoblasts, osteocytes, and activated T lymphocytes [10]. The soluble form of the protein (sRANKL) is generated by either proteolytic processing by matrix metalloproteases or alternative mRNA splicing [11]. RANKL binds to RANK on the surface of osteoclasts and osteoclast precursors, leading to their differentiation and activation [12,13]. In the 5T2MM mouse model of multiple myeloma, MPCs overexpress RANKL, which directly promotes the formation of osteoclasts and osteolytic lesions [14]. Terpos et al. first reported elevated serum levels of sRANKL in MM patients and suggested that RANKL/OPG system is essential for the development of MBD [15]. The early involvement of RANKL signaling was observed in patients with monoclonal gammopathy of undetermined significance (MGUS), where circulating sRANKL levels were higher than in controls but still lower than in patients with symptomatic MM [16]. Serum RANKL was significantly increased in advanced clinical stages and high grade MBD of MM patients [17,18]. Therefore, inhibition of the RANK/RANKL pathway is an important target for the prevention of SRE in MM. Denosumab, a fully human monoclonal anti-RANKL antibody, has been in use for more than ten years in clinical practice [19].

Another protein associated with bone metabolism is the extracellular matrix (ECM) protein periostin, which is produced primarily by stromal mesenchymal cells and is mainly expressed in the periosteum [20,21]. In addition to its structural role in the bone matrix, under physiological conditions, periostin stimulates osteoblastic function and bone formation activating the Wnt/β-catenin signaling pathway [22]. Under pathological conditions, high serum periostin is associated with an increased risk of fractures in postmenopausal women [23,24], and in ankylosing spondylitis [25]. Many studies have reported elevated periostin expression in primary tumors and metastatic lesions, e.g., colorectal cancer and liver metastatic lesions [26,27,28,29,30,31]. There are very scarce data concerning periostin implication in MM. Terpos et al. first demonstrated high periostin in the supernatant of six myeloma cell lines. Beside the experimental data, the authors investigated periostin in clinical settings and found the progressive elevation of its levels as the disease evolves from MGUS, SMM (smoldering multiple myeloma), and symptomatic MM [32]. Revealing the role of periostin in the pathogenesis of MBD, target therapy with anti-periostin antibodies might be a promising new treatment strategy.

Another abundant non-collagenous ECM protein that is an important component of the BM microenvironment is osteopontin (OPN) [33]. It is expressed by a wide variety of cell types such as bone cells, neurons, epithelial cells, pericytes, fibroblasts, hepatocytes, tubular cells, vascular smooth muscle cells, and immune cells [34]. It is involved in a number of physiological and pathological processes, including bone remodeling, angiogenesis, immune-regulation, inflammation, wound healing, atherosclerosis, and tumor progression [33,35,36,37,38]. In models with human MM cell lines, Abe et al. demonstrated a cell–cell contact-mediated mechanism of OC induced MPC growth and survival, partially dependent on IL-6 and OPN. A vicious cycle is supposed to be formed, in which the production of OPN along with IL-6 from OCs recruited around MPCs contribute to extensive bone destruction and MPC expansion [39]. Various studies have found significantly elevated plasma OPN levels in symptomatic MM patients compared to MGUS patients, patients in remission and healthy controls [40,41,42]. OCs-derived osteopontin and VEGF from MPCs cooperatively enhance angiogenesis and also induce osteoclastogenic activity by vascular endothelial cells [43].

The aim of the present study was to explore circulating levels of sRANKL, periostin, and osteopontin in newly diagnosed MM (NDMM) patients at diagnosis and in the course of their treatment, including autologous stem cell transplantation (ASCT), and to assess possible correlations with laboratory data, disease stage, and MBD.

## 2. Materials and Methods

### 2.1. Study Population and Study Design

A total of 41 NDMM patients were enrolled in this single-center non-interventional prospective study, carried out between June 2021 and December 2022. Selected patients met the following inclusion criteria: 18 to 80 years of age; and diagnosed according to the Revised International Myeloma Working Group criteria [44]. Patients with autoimmune or other malignant diseases and treated with bone affecting drugs during the last six months were excluded from the study. The disease stage was determined according to the International Staging System, ISS [45]. The study design was explained in detail in [46]. In brief, whole-body low-dose computed tomography (WBLDCT) imaging was used for the assessment of MBD, defined by osteolytic lesions, pathological fractures, vertebral collapse, and soft-tissue involvement. All patients were treated using the standard VCD chemotherapeutic protocol. In addition, patients with a glomerular filtration rate of more than 30 mL/min/1.73 m^2^ received biphosphonates. International Myeloma Working Group (IMWG) consensus criteria for response were used to evaluate response to treatment [47]. After they completed four cycles of VCD treatment, the patients were divided into two groups: patients below 65 years and with a complete response (CR) and very good partial response (VGPR) proceeded to ASCT; patients with stable disease (SD) or partial response (PR) continued with another four cycles of VCD. Patients with progressive disease in the course of treatment dropped out from the study. Four time points for the evaluation of the variations in the tested biochemical markers were selected: before treatment (time point T0); after four cycles of VCD treatment (time point T1); after eight cycles of VCD treatment (time point T2); three months after ASCT (time point TA).

Thirty-three healthy age- and gender-matched control subjects without any chronic inflammatory, autoimmune, or neoplastic disease or bisphosphonate and other bone-affecting drug medication six months before starting the survey were included in the study.

### 2.2. Methods

Serum sRANKL, periostin, and osteopontin were assayed by commercial ELISA (Sunredbio, Shanghai, China), strictly following the manufacturer’s instructions. Hematological and biochemical parameters as well as morphological evaluations of bone marrow aspirate smears were routinely monitored.

### 2.3. Statistical Analysis

Results were presented as the mean ± standard deviation (SD), median, and interquartile range (IQR: 25–75-th percentile), or number (*n*) and percentage (%), as appropriate. Descriptive statistics, the unpaired Student’s *t*-test, and the Mann–Whitney U-test for comparison of Gausian or non-Gausian distributed interval data were used. Chi-square analysis was used to evaluate the frequency distribution according to gender. The relationship between continuous variables was evaluated by Spearman’s correlation analysis, and if a significant relation was found, a linear regression analysis was applied to test the associations of sRANKL, periostin, and osteopontin with routine laboratory parameters. Receiver operating characteristic (ROC) analysis was performed for evaluation the predictive power of serum sRANKL, periostin, and osteopontin. All statistical analyses were two-tailed and statistical significance was considered at *p* < 0.05. Data analysis was performed using GraphPad Prism v. 8.0.1, San Diego, CA, USA.

## 3. Results

The baseline demographic and clinical characteristics, as well some results from the routine laboratory testing of MM patients enrolled in the study are provided in detail in [46] and in Supplementary Table S1.

### 3.1. Baseline Serum Levels of sRANKL, Periostin, and Osteopontin in Newly Diagnosed Multiple Myeloma Patients

**Figure 1:**
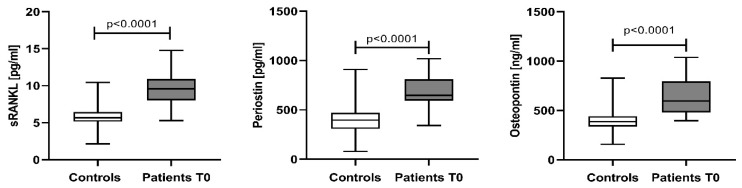
Serum levels of sRANKL, periostin, and osteopontin in controls and patients at baseline. Data are presented as medians and minimal and maximal values. Statistical significance was indicated at *p* < 0.05.

The median serum levels and IQR for sRANKL, periostin, and osteopontin in NDMM patients at baseline (T0) were: 9.59 pg/mL (8.03–10.92), 648.4 pg/mL (594.4–809.9), and 596.0 ng/mL (479.7–793.8), respectively, significantly higher than those in controls: 5.67 pg/mL (5.15–6.44) for sRANKL, 396.9 pg/mL (308.6–471.9) for periostin, and 387.0 ng/mL (335.9–441.9) for osteopontin (Figure 1).

**Figure 2:**
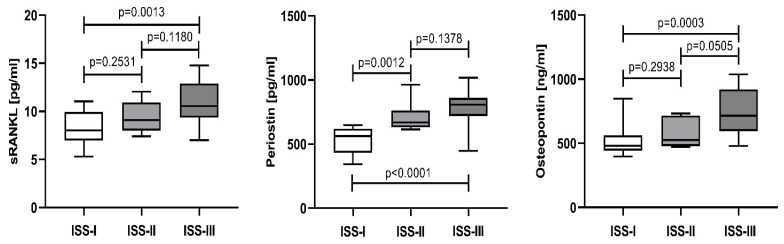
Comparison of sRANKL, periostin, and osteopontin serum levels in NDMM patients at baseline (T0) and stratified according to the ISS staging system. Data are presented as medians and minimal and maximal values. Statistical significance was indicated at *p* < 0.05.

The serum levels of sRANKL, periostin, and osteopontin gradually increased with the disease stage. The median and IQR for sRANKL in patients in ISS-I, ISS-II, and ISS-III were 8.03 pg/mL (6.97–9.94), 9.09 pg/mL (8.02–10.92), and 10.55 pg/mL (9.36–12.89), respectively. The same phenomenon was observed for periostin: the medians and their IQR for ISS-I, ISS-II, and ISS-III were 563.2 pg/mL (432.8–620.6), 669.0 pg/mL (632.3–761.4), and 807.6 pg/mL (719.6–860.5), respectively, as well as for osteopontin: 480.0 ng/mL (442.8–562.0) in ISS-I stage, 526.2 ng/mL (477.7–715.0) in ISS-II stage and 715.0 ng/mL (596.0–920.1) in ISS-III stage. A statistical difference was reached between ISS-I and ISS-III for all three parameters: for sRANKL—*p* = 0.0013, for periostin—*p* < 0.0001, and for osteopontin—*p* = 0.0003. A significant difference between ISS-I and ISS-II was detected only for periostin (*p* = 0.0012), as well for osteopontin between ISS-II and ISS-III—*p* = 0.0505 (Figure 2).

**Figure 3:**
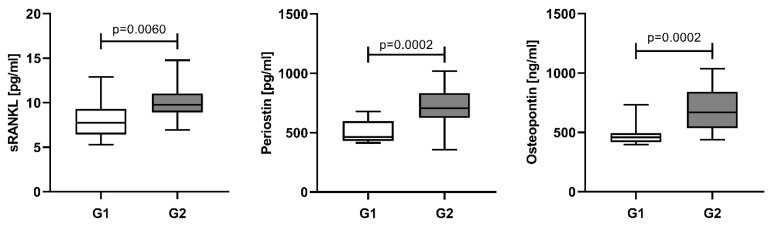
Serum levels of sRANKL, periostin and osteopontin in NDMM patients, stratified by the number of bone lesions. Data are presented as medians and minimal and maximal values. Statistical significance was indicated at *p* < 0.05.

Lytic bone lesions (≥5 mm) on WBLDCT have been integrated into the diagnostic criteria of the IMWG [48]. According to the number of bone lesions the patients were divided into two groups: G1—patients with 0 to 3 osteolytic lesions, and G2—patients with more than three lesions and/or bone fractures. The serum levels of the assessed proteins were significantly higher in the G2 group: sRANKL 9.76 pg/mL (IQR 8.90–11.04); periostin 706.8 pg/mL (IQR 624.0–833.6); osteopontin 669.8 ng/mL (IQR 535.6–843.1), compared to G1 group: sRANKL 7.75 pg/mL (IQR 6.44–9.29); periostin 463.8 pg/mL (IQR 431.0–596.4); osteopontin 669.8 ng/mL (IQR 535.6–843.1); 460.0 ng/mL (IQR 419.1–494.8) (Figure 3).

**Figure 4:**
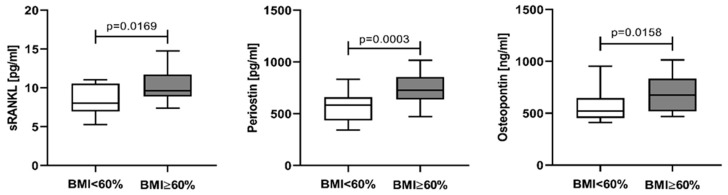
Serum levels of sRANKL, periostin, and osteopontin in NDMM patients, stratified according to BMI by MPCs. Data are presented as medians and minimal and maximal values. Statistical significance was indicated at *p* < 0.05.

The impact of tumor burden on sRANKL, periostin, and osteopontin serum levels was evaluated by dividing the patients into two groups according to the BMI by MPCs using a cut-off of 60% [49]. Twenty-four NDMM patients (58.54%) were with BMI ≥ 60% MPCs and their serum levels of sRANKL (median 9.63 pg/mL, IQR 8.90–11.73), periostin (median 726.9 pg/mL, IQR 639.9–855.1), and osteopontin (median 676.7 ng/mL, IQR 517.7–834.3) were significantly higher than the corresponding values in patients with BMI < 60% MPCs: 8.03 pg/mL (IQR 6.96–10.55) for sRANKL, 585.6 pg/mL (IQR 436.3–660.1) for periostin, and 521.2 ng/mL (IQR 453.9–646.5) for osteopontin (Figure 4).

**Figure 5:**
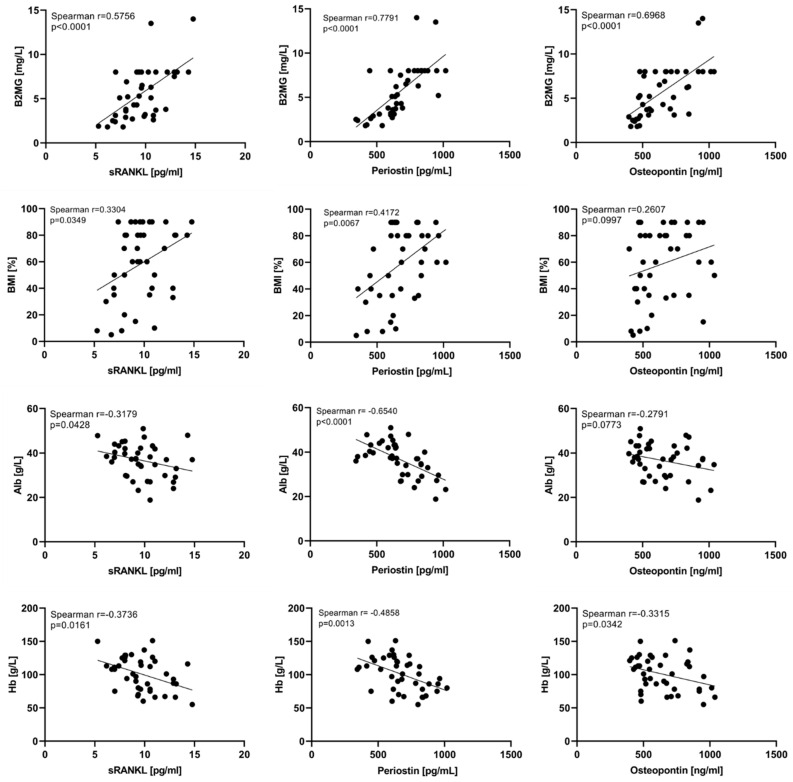
Spearman correlations of sRANKL, periostin, and osteopontin with B2MG, BMI, albumin, and Hb. Β2MG—β2 microglobulin, BMI—bone marrow infiltration by MPCs, Alb—albumin, Hb—hemoglobin. Statistical significance was indicated at *p* < 0.05.

Spearman correlation analysis was performed to evaluate the relationship between the tested bone markers and some routine laboratory parameters. A statistically significant (*p* < 0.0001) strong positive correlation with B2MG was found for periostin (r = 0.779), osteopontin (r = 0.697), and for sRANKL (r = 0.576). Bone marrow infiltration (BMI) by MPCs correlates significantly with sRANKL (r = 0.330, *p* = 0.0349) and periostin (r = 0.417, *p* = 0.0067) serum levels, while for osteopontin we did not find such a significant relation (r = 0.261, *p* = 0.0997). Spearman’s correlation analysis with creatinine was also performed to evaluate a possible relationship between the tested bone parameters and the renal function of MM patients. A significant positive correlation was found for the tested three parameters, stronger for periostin and osteopontin. Only periostin revealed a significant positive correlation with total protein. A significant negative association with albumin, as well with hemoglobin was found for all examined parameters (Figure 5 and Supplementary Table S2).

### 3.2. Variation of sRANKL, Periostin, and Osteopontin Serum Levels in the Course of Treatment and According to the Response to Therapy

**Figure 6:**
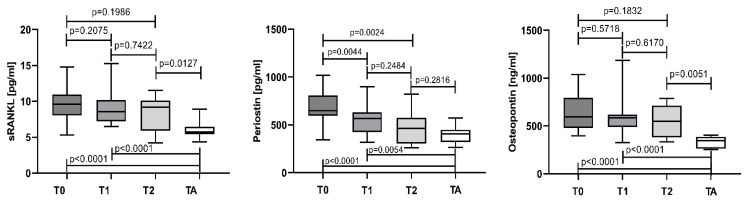
sRANKL, periostin, and osteopontin assessed at different time points.

The medians and corresponding IQR of sRANKL, periostin, and osteopontin serum levels at different time points in the course of treatment are given in Supplementary Table S3. Serum levels for sRANKL, periostin, and osteopontin gradually decreased in the course of treatment. A significant reduction in periostin was already observed at time point T1, while sRANKL and osteopontin were non-significantly lower than the baseline values (T0). For patients, not eligible for transplantation, the periostin levels continued to decrease and remained significantly lower at time point T2 compared to the T0 values (*p* = 0.0024), while the sRANKL and osteopontin changes continued to not show any significant reduction. Patients who underwent ASCT, showed the lowest values for all measured proteins at TA, significantly different from those in T0 and T1 (Figure 6).

Examining the tested parameter changes in the course of treatment in relation to the control values, we found significantly higher serum periostin at T1 (*p* = 0.0005), while at T2 and TA it almost reached the control levels (*p* = 0.1899 and *p* > 0.9999, respectively). The same phenomenon was observed for sRANKL and osteopontin only at the TA time point (*p* = 0.5614 and *p* = 0.0996, respectively).

The response to therapy at all treatment points is summarized in Table 1.

Table 2 represents the changes of sRANKL, periostin, and osteopontin serum levels depending on treatment response.

**Figure 7:**
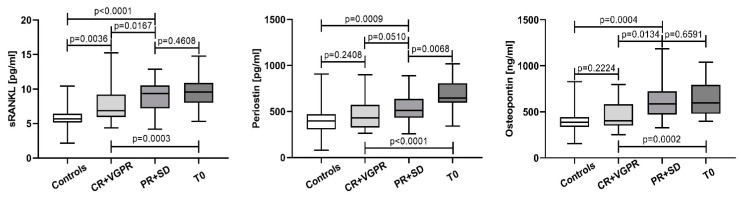
sRANKL, periostin, and osteopontin according to the treatment response. Data are presented as medians and minimal and maximal values. Statistical significance was indicated at *p* < 0.05.

Patients with a CR and VGPR revealed significantly lower levels for the tested parameters vs. baseline levels (T0). In addition, periostin and osteopontin almost reached the control values, while sRANKL remained significantly higher (*p* = 0.0036). On the other hand, sRANKL and osteopontin remained unchanged compared to T0 in PR and SD patients, while periostin was significantly decreased. Regarding the controls, the patients from this group revealed significantly higher values for all measured proteins (Figure 7).

### 3.3. Predicting Power of sRANKL, Periostin, and Osteopontin

**Figure 8:**
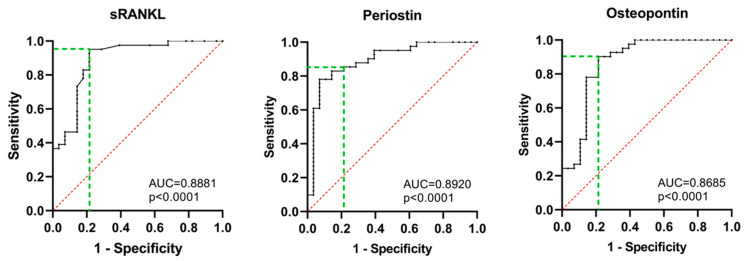
Diagnostic performance of sRANKL, periostin, and osteopontin assessed by ROC curve analysis. ROC curve—receiver operating characteristic curve, AUC—area under the curve. The green dotted line represents the settings for finding the optimal diagnostic cut-off values of the tested parameters. Statistical significance was indicated at *p* < 0.05.

ROC analysis was performed to evaluate the diagnostic utility of the tested parameters for MBD assessment in NDMM patients (Table 3 and Figure 8).

The area under the ROC curve for each of the tested parameters is above 0.85 (*p* < 0.0001), revealing a relatively high distinguishing capacity between positive and negative cases of MBD in NDMM patients. All tested proteins revealed high diagnostic sensitivity and specificity at the most appropriate cut-off value.

Multiple linear regression analysis was used to estimate the relationship between each one of tested parameters as a dependent variable and several independent variables with which a significant correlation was established. For sRANKL linear regression was calculated with B2M, BMI, Hb, albumin, and creatinine. The regression model was statistically valid with F = 5.546, *p* = 0.0007 and adjusted R2 = 0.3624. Between the independent variables, only B2M revealed significant association (β = 0.5538, *p* = 0.0017) with sRANKL. The regression model performed for periostin included B2M, BMI, Hb, albumin, and creatinine and was also statistically significant (F = 12.27, *p* < 0.0001 and adjusted R2 = 0.5849). B2M, BMI, and albumin revealed significant associations (β = 25.04, *p* = 0.0164; β = 1.585, *p* = 0.0248 and β = −7.562, *p* = 0.0145, respectively). A statistically significant regression model was constructed for osteopontin and B2M, Hb, and creatinine (F = 10.73, *p* < 0.0001 and adjusted R2 = 0.4218). Only B2M revealed a significant association (β = 3.706, *p* = 0.0007) with osteopontin.

## 4. Discussion

The pathogenesis of MBD is multifactorial. Interactions of MPCs with various cell types such as lymphocytes, endothelial and stromal cells, as well as components of the extracellular bone marrow microenvironment play a crucial role in the development of MBD. In the last two decades, several cytokines and ECM proteins have been evaluated as biomarkers for the assessment of MBD. We analyzed the serum levels of sRANKL, periostin, and osteopontin in NDMM patients depending on the clinical stage and grade of MBD and their variations in the course of therapy to evaluate their potential as additional biomarkers for the assessment of MBD.

### 4.1. Baseline Serum Levels of sRANKL, Periostin, and Osteopontin in Newly Diagnosed Multiple Myeloma Patients

The role of sRANKL as a potent osteoclast activating factor is well established in animal and in vitro models [14,50,51,52]. mRNA and soluble RANKL have been observed in samples from MM patients, but not in chronic lymphocytic leukemia, indicating a unique requirement for RANKL in multiple myeloma [53]. In our study the sRANKL serum levels in NDMM patients at baseline were significantly higher than those in controls. In agreement with our results are the findings of most previous studies, which have demonstrated significantly higher values for sRANKL in NDMM patients vs. control subjects [15,16,17,54]. A few reports did not find a statistical difference in the serum levels of sRANKL in NDMM patients compared to controls, and no differences were observed with respect to disease stage and bone lesions [55,56]. The inconsistency of the abovementioned results could be explained by the different ELISA kits used for sRANKL determination. It is not surprising that RANKL, as a potent osteoclast activator, increases with osteoclast bone lesions as well as with the disease stage. In our study lower RANKL levels were associated with a small number of osteolytic lesions (<3, G1), whereas higher RANKL was related to advanced bone disease (G2). Similar relationships were observed for circulating RANKL and disease stage, increased in ISS-2 and ISS-3, and lower in ISS-1. Our findings agree with other studies reporting a significant increase of serum RANKL with advanced disease stage, grade of bone disease, and factors of disease activity such as B2MG and BMI [15,16,17,18,54,57].

The role of periostin in the pathogenesis of MM is poorly understood. In an experimental study it was suggested that fibroblast-like cells from the BM microenvironment are implicated in ECM remodeling and lead to the upregulation of various matrix proteins, including periostin, thus creating a favorable environment for MPC development. The upregulation takes place already at the level of MGUS and is more pronounced in MM [58]. Data from clinical studies regarding periostin are really scarce. Terpos et al. were the first to show significantly increased serum levels of periostin in NDMM and relapsed patients compared not only to healthy subjects, but also to MGUS and SMM patients. The authors found that serum periostin strongly correlated with B2MG and ISS-stages. The established correlations of periostin (positive with CTX, a bone resorption marker, and negative with bone alkaline phosphatase, a bone formation marker) suggest the role of periostin in the development of MBD. Moreover, patients with pathological fractures had markedly elevated periostin both in serum and BM plasma in comparison with all others. Patients with osteolytic lesions had significantly higher periostin in BM, but not in serum compared to those without osteolytic lesions [32]. Consistent with these data are our results which demonstrate a significant increase in serum periostin levels in NDMM patients vs. controls. We also established that periostin levels progressively increased both with the disease stages and the severity of bone involvement. In contrast with the finding of Terpos et al. we found significant differences in periostin serum levels in the G2 group vs. G1 group. The discordance may be due to the fact that patients with pathological fractures were also included in the G2 group. Also findings a moderate to strong positive correlation of serum periostin with B2MG, BMI, creatinine, and TP, as well as a negative correlation with albumin and Hb, all of which are associated with disease progression, we suggest the important role of periostin for MPC invasion in the BM and for MBD.

OPN, as the most abundant non-collagenous protein in the bone ECM, is an integral part of the BM microenvironment, where it regulates matrix interactions and cell adhesion [59]. For bone resorption, OPN-mediated attachment of OCs to the bone surface represents a crucial step. The role of OPN in MM is better studied than that of periostin, but different results have been reported. In most studies, the baseline OPN levels in symptomatic patients were significantly higher than those of controls [40,41,42,60,61]. We have also obtained similar results. On the contrary, Kang et al. did not find a significant difference in OPN levels between MM patients and healthy controls [62]. Moreover, in a recent study, even significantly lower OPN levels were found in stage I MM patients [63]. This discrepancy is probably due to the small patient sample, poorly defined patients’ disease stages, and the ELISA method used for osteopontin determination. Our data revealing statistically higher levels in ISS-III patients compared to ISS-I patients, as well in the G2 vs. G1 group, confirm the results of several studies that OPN levels reflect the progression of MM and MBD well [42,61,64]. In addition, we proved that patients with BMI ≥ 60% MPCs had significantly higher levels of OPN than those with BMI < 60% MPCs. OPN is produced not only by osteoclasts, but also by MPCs and in turn elevated OPN stimulates MPCs proliferation and exerts a proangiogenic effect on BM blood vessels [39,65,66], thus forming a vicious cycle of the self-maintenance and progression of the tumor process. Sfiridaki et al. reported a significant correlation between serum OPN and disease infiltration, as well with serum vascular endothelial growth factor (VEGF) and BM microvessel density (MVD) as markers of BM neovascularization [64]. In an Interesting in vitro study Robbiani et al. showed that high OPN expression correlates inversely with bone disease, explained by presence of maf translocations [67]. In the present study, the association of OPN with tumor burden was also proven by a strong positive correlation with B2MG and a weak negative correlation with albumin and hemoglobin. Similar results were reported by other researchers [41,60,61,64].

### 4.2. Variation of sRANKL, Periostin, and Osteopontin Serum Levels in the Course of Treatment and According to the Response to Therapy

Bortezomib-based regimens are most frequently used for MM [68]. In addition to an antimyeloma effect, Bortezomib (Velcade) induces mesenchyme stem cells to preferentially undergo osteoblastic differentiation and suppresses osteoclasts, ultimately leading to bone formation [69,70].

In the first clinical study concerning the effect of Bortezomib administration on bone remodeling markers, Terpos et al. have shown significantly reduced serum levels of sRANKL, DKK-1, CTX, and TRACP-5b after four cycles of therapy, irrespective of treatment response, thus leading to the normalization of bone remodeling in relapsed myeloma patients [71]. In several other clinical studies bortezomib-based therapies also have led to a significant reduction of serum RANKL [64,69]. Our results are consistent with the abovementioned findings showing a gradual decrease of sRANKL in the course of treatment. Moreover, we found significantly lower levels of this cytokine in NDMM patients with a CR and VGPR vs. those with a PR and SD.

In our study, circulating periostin showed a strong association with the course of treatment. In the different time points of chemotherapy or after transplantation, its serum levels were significantly lower than those at diagnosis. Moreover, the patients with a CR and VGPR revealed significantly lower periostin levels compared to those with a PR and in stable disease. To our knowledge, we are the first to demonstrate that circulating periostin reflects the effectiveness of therapy.

In comparison with sRANKL, considerably less data are available in the literature regarding the effect of therapy on serum OPN levels. Sfiridaki et al. have shown significantly lower OPN after chemotherapy [64]. Minarik et al. found not only lower post-chemotherapeutic OPN levels, but also significantly lower levels in patients with a deep response in comparison with initial OPN levels [42]. In our study, we examined OPN level variations in the course of treatment and found a gradual decrease with the lowest levels achieved 3 months after ASCT.

According to the ROC analysis, the three biomarkers show similar diagnostic reliability (AUC approximately 0.9, *p* < 0.0001). Depending on the ISS-stage, serum periostin and osteopontin differentiate the intermediate ISS-II, which was not observed for sRANKL (Figure 2). According to treatment response, periostin most adequately reflects the treatment effect. In patients with a CR + VGPR, periostin serum levels decrease and almost reach control levels. In patients with a PR + SD, they remain elevated vs. the controls and a CR + VGPR but are lower than baseline levels. Based on these findings, we could hypothesize that serum periostin might be the most sensitive indicator among the tested parameters. Further studies involving larger cohorts should be conducted to confirm this hypothesis.

As these ECM proteins have been very scarcely studied in clinical settings, we consider the monitoring of OPN, PON, and RANKL serum levels in NDMM patients and the effect of therapy on their serum levels to constitute the strengths of our study. There are also some limitations in our study. One limitation is the small number of studied patients. Another is that we did not examine the changes in osteoprotegerin (OPG) levels in NDMM patients and its interrelations with the proteins that we tested, especially with sRANKL.

## 5. Conclusions

MBD has a significant impact on patient quality of life. The discovery and validation of sensitive serum bone markers would aid in the prediction of skeletal complications and their early treatment, improving patient outcomes and reducing the burden on healthcare systems. The present study demonstrates activated osteoclastogenesis, evident by elevated levels of bone resorption cytokines sRANKL, periostin, and osteopontin in NDMM. Moreover, their increased levels strongly correlate with the MM stage and the grade of MBD. Studying the dynamics of these proteins, we demonstrated a significant decrease during treatment, more pronounced after ASCT. In the context of these findings, sRANKL, periostin, and osteopontin could be promising markers for monitoring MBD in MM and the effect of treatment.

## Figures and Tables

**Table 1:** Frequency distribution according to treatment response.

Time Point	CR + VGPR (n/N, %)	PR + SD (n/N, %)	PD (n/N, %)
T1	10/22 (45.5)	11/22 (50.0)	1/22 (4.5)
T2	3/11 (27.3)	8/11 (72.7)	0
TA	10/10 (100.0)	0	0

**Table 2:** Serum levels of sRANKL, periostin, and osteopontin according to treatment response.

Parameter	CR + VGPR Median (IQR)	PR + SD Median (IQR)	*p* Value
sRANKL [pg/mL]	6.88 (5.92–9.21)	9.36 (7.20–10.55)	*p* = 0.0167
Periostin [pg/mL]	432.0 (594.4–809.9)	511.9 (432.7–638.1)	*p* = 0.0134
Osteopontin [ng/mL]	403.8 (350.2–583.4)	586.9 (471.4–724.1)	*p* = 0.0510

CR—complete response; VGPR—very good partial response; PR—partial response; SD—stable disease.

**Table 3:** Diagnostic utility of the tested parameters evaluated by ROC curve analysis.

Parameter	AUC, *p* Value	Cut-off Value	Diagnostic Sensitivity % (CI)	Diagnostic Specificity % (CI)	Likelihood Ratio
sRANKL	0.8881, *p* < 0.0001	6.572 pg/mL	95.0 (83.9–99.1)	78.6 (60.5–89.8)	4.439
Periostin	0.8920, *p* < 0.0001	473.1 pg/mL	85.4 (71.6–93.1)	78.6 (60.5–89.8)	3.984
Osteopontin	0.8685, *p* < 0.0001	447.1 ng/mL	90.2 (77.5–96.1)	78.6 (60.5–89.8)	4.211

ROC—receiver operator characteristics; AUC—area under the ROC curve; CI—confidential interval.

## Data Availability

The data presented in this study are available in this article.

## Supplementary Materials

The following supporting information can be downloaded at: https://www.mdpi.com/article/10.3390/cancers15235562/s1. Table S1: Patients’ demographic and clinical characteristics at baseline. Table S2: Correlations of sRANKL, periostin and osteopontin with some laboratory parameters; Table S3: Serum values of sRANKL, Periostin and Osteopontin, measured at different time points.

## References

[1] Padala S.A., Barsouk A., Barsouk A., Rawla P., Vakiti A., Kolhe R., Kota V., Ajebo G.H. (2021). Epidemiology, Staging, and Management of Multiple Myeloma. Med. Sci..

[2] Rajkumar S.V. (2019). Multiple Myeloma: Every Year a New Standard?. Hematol. Oncol..

[3] Terpos E., Ntanasis-Stathopoulos I., Gavriatopoulou M. (2018). Pathogenesis of Bone Disease in Multiple Myeloma: From Bench to Bedside. Blood Cancer J..

[4] Hagiwara M., Panjabi S., Delea T., Yucel E., Fonseca R. (2020). Burden of Disease Progression in Patients with Multiple Myeloma in the US. Leuk. Lymphoma.

[5] Kumar R., Godavarthy P.S., Krause D.S. (2018). The Bone Marrow Microenvironment in Health and Disease at a Glance. J. Cell Sci..

[6] Bernstein Z.S., Kim E.B., Raje N. (2022). Bone Disease in Multiple Myeloma: Biologic and Clinical Implications. Cells.

[7] Mukkamalla S.K.R., Malipeddi D. (2021). Myeloma Bone Disease: A Comprehensive Review. Int. J. Mol. Sci..

[8] Gau Y.-C., Yeh T.-J., Hsu C.-M., Hsiao S.Y., Hsiao H.-H. (2022). Pathogenesis and Treatment of Myeloma-Related Bone Disease. Int. J. Mol. Sci..

[9] Mansour A., Wakkach A., Blin-Wakkach C. (2017). Emerging Roles of Osteoclasts in the Modulation of Bone Microenvironment and Immune Suppression in Multiple Myeloma. Front. Immunol..

[10] Kim J.-M., Lin C., Stavre Z., Greenblatt M.B., Shim J.-H. (2020). Osteoblast-Osteoclast Communication and Bone Homeostasis. Cells.

[11] Tobeiha M., Moghadasian M.H., Amin N., Jafarnejad S. (2020). RANKL/RANK/OPG Pathway: A Mechanism Involved in Exercise-Induced Bone Remodeling. BioMed Res. Int..

[12] Raje N.S., Bhatta S., Terpos E. (2019). Role of the RANK/RANKL Pathway in Multiple Myeloma. Clin. Cancer Res..

[13] Wu X., Li F., Dang L., Liang C., Lu A., Zhang G. (2020). RANKL/RANK System-Based Mechanism for Breast Cancer Bone Metastasis and Related Therapeutic Strategies. Front. Cell Dev. Biol..

[14] Buckle C.H., De Leenheer E., Lawson M.A., Yong K., Rabin N., Perry M., Vanderkerken K., Croucher P.I. (2012). Soluble Rank Ligand Produced by Myeloma Cells Causes Generalised Bone Loss in Multiple Myeloma. PLoS ONE.

[15] Terpos E., Szydlo R., Apperley J.F., Hatjiharissi E., Politou M., Meletis J., Viniou N., Yataganas X., Goldman J.M., Rahemtulla A. (2003). Soluble Receptor Activator of Nuclear Factor κB Ligand–Osteoprotegerin Ratio Predicts Survival in Multiple Myeloma: Proposal for a Novel Prognostic Index. Blood.

[16] Politou M., Terpos E., Anagnostopoulos A., Szydlo R., Laffan M., Layton M., Apperley J.F., Dimopoulos M., Rahemtulla A. (2004). Role of Receptor Activator of Nuclear Factor-kappa B Ligand (RANKL), Osteoprotegerin and Macrophage Protein 1-alpha (MIP-1a) in Monoclonal Gammopathy of Undetermined Significance (MGUS). Br. J. Haematol..

[17] Goranova-Marinova V., Goranov S., Pavlov P., Tzvetkova T. (2007). Serum Levels of OPG, RANKL and RANKL/OPG Ratios in Newly-Diagnosed Patients with Multiple Myeloma. Clinical Correlations. Haematologica.

[18] Jakob C., Goerke A., Terpos E., Sterz J., Heider U., Kühnhardt D., Ziefle S., Kleeberg L., Mieth M., Von Metzler I. (2009). Serum Levels of Total-RANKL in Multiple Myeloma. Clin. Lymphoma Myeloma.

[19] Cadieux B., Coleman R., Jafarinasabian P., Lipton A., Orlowski R.Z., Saad F., Scagliotti G.V., Shimizu K., Stopeck A. (2022). Experience with Denosumab (XGEVA^®^) for Prevention of Skeletal-Related Events in the 10 Years after Approval. J. Bone Oncol..

[20] Wang Z., An J., Zhu D., Chen H., Lin A., Kang J., Liu W., Kang X. (2022). Periostin: An Emerging Activator of Multiple Signaling Pathways. J. Cell Commun. Signal..

[21] Cobo T., Viloria C.G., Solares L., Fontanil T., González-Chamorro E., De Carlos F., Cobo J., Cal S., Obaya A.J. (2016). Role of Periostin in Adhesion and Migration of Bone Remodeling Cells. PLoS ONE.

[22] Bonnet N., Garnero P., Ferrari S. (2016). Periostin Action in Bone. Mol. Cell. Endocrinol..

[23] Kim B.-J., Lee S.H., Koh J.-M. (2020). Potential Biomarkers to Improve the Prediction of Osteoporotic Fractures. Endocrinol. Metab..

[24] Rousseau J.C., Sornay-Rendu E., Bertholon C., Chapurlat R., Garnero P. (2014). Serum Periostin Is Associated With Fracture Risk in Postmenopausal Women: A 7-Year Prospective Analysis of the OFELY Study. J. Clin. Endocrinol. Metab..

[25] Sakellariou G.T., Anastasilakis A.D., Bisbinas I., Oikonomou D., Gerou S., Polyzos S.A., Sayegh F.E. (2015). Circulating Periostin Levels in Patients with AS: Association with Clinical and Radiographic Variables, Inflammatory Markers and Molecules Involved in Bone Formation. Rheumatology.

[26] Kapoor S. (2014). Periostin and Its Emerging Role in Systemic Carcinogenesis. Osteoporos. Int..

[27] Underwood T.J., Hayden A.L., Derouet M., Garcia E., Noble F., White M.J., Thirdborough S., Mead A., Clemons N., Mellone M. (2015). Cancer-associated Fibroblasts Predict Poor Outcome and Promote Periostin-dependent Invasion in Oesophageal Adenocarcinoma. J. Pathol..

[28] Wu G., Wang X., Zhang X. (2013). Clinical Implications of Periostin in the Liver Metastasis of Colorectal Cancer. Cancer Biother. Radiopharm..

[29] Kim G.-E., Lee J.S., Park M.H., Yoon J.H. (2017). Epithelial Periostin Expression Is Correlated with Poor Survival in Patients with Invasive Breast Carcinoma. PLoS ONE.

[30] Tilman G., Mattiussi M., Brasseur F., Van Baren N., Decottignies A. (2007). Human Periostin Gene Expression in Normal Tissues, Tumors and Melanoma: Evidences for Periostin Production by Both Stromal and Melanoma Cells. Mol. Cancer.

[31] Sasaki H., Auclair D., Kaji M., Fukai I., Kiriyama M., Yamakawa Y., Fujii Y., Chen L.B. (2001). Serum Level of the Periostin, a Homologue of an Insect Cell Adhesion Molecule, in Thymoma Patients. Cancer Lett..

[32] Terpos E., Christoulas D., Kastritis E., Bagratuni T., Gavriatopoulou M., Roussou M., Papatheodorou A., Eleutherakis-Papaiakovou E., Kanellias N., Liakou C. (2016). High Levels of Periostin Correlate with Increased Fracture Rate, Diffuse MRI Pattern, Abnormal Bone Remodeling and Advanced Disease Stage in Patients with Newly Diagnosed Symptomatic Multiple Myeloma. Blood Cancer J..

[33] Wai P.Y., Kuo P.C. (2008). Osteopontin: Regulation in Tumor Metastasis. Cancer Metastasis Rev..

[34] Kaleta B., Boguska A. (2017). Sildenafil, a Phosphodiesterase Type 5 Inhibitor, Downregulates Osteopontin in Human Peripheral Blood Mononuclear Cells. Arch. Immunol. Ther. Exp..

[35] Denhardt D.T., Mistretta D., Chambers A.F., Krishna S., Porter J.F., Raghuram S., Rittling S.R. (2003). Transcriptional Regulation of Osteopontin and the Metastatic Phenotype: Evidence for a Ras-Activated Enhancer in the Human OPN Promoter. Clin. Exp. Metastasis.

[36] Cho H.-J., Cho H.-J., Kim H.-S. (2009). Osteopontin: A Multifunctional Protein at the Crossroads of Inflammation, Atherosclerosis, and Vascular Calcification. Curr. Atheroscler. Rep..

[37] Kim J.-M., Kim M.Y., Lee K., Jeong D. (2016). Distinctive and Selective Route of PI3K/PKCα-PKCδ/RhoA-Rac1 Signaling in Osteoclastic Cell Migration. Mol. Cell. Endocrinol..

[38] Walker C.G., Dangaria S., Ito Y., Luan X., Diekwisch T.G.H. (2010). Osteopontin Is Required for Unloading-Induced Osteoclast Recruitment and Modulation of RANKL Expression during Tooth Drift-Associated Bone Remodeling, but Not for Super-Eruption. Bone.

[39] Abe M., Hiura K., Wilde J., Shioyasono A., Moriyama K., Hashimoto T., Kido S., Oshima T., Shibata H., Ozaki S. (2004). Osteoclasts Enhance Myeloma Cell Growth and Survival via Cell-Cell Contact: A Vicious Cycle between Bone Destruction and Myeloma Expansion. Blood.

[40] Saeki Mima Y.T., Ishii T., Ogata A., Kobayashi H., Ohshima S., Ishida T., Tabunoki Y., Kitayama H., Mizuki M., Katada Y. (2003). Enhanced Production of Osteopontin in Multiple Myeloma: Clinical and Pathogenic Implications. Br. J. Haematol..

[41] Standal T., Hjorth-Hansen H., Rasmussen T., Dahl I.M.S., Lenhoff S., Brenne A.-T., Seidel C., Baykov V., Waage A., Børset M. (2004). Osteopontin Is an Adhesive Factor for Myeloma Cells and Is Found in Increased Levels in Plasma from Patients with Multiple Myeloma. Haematologica.

[42] Minarik J., Pika T., Bacovsky J., Petrova P., Langova K., Scudla V. (2012). Prognostic Value of Hepatocyte Growth Factor, Syndecan-1, and Osteopontin in Multiple Myeloma and Monoclonal Gammopathy of Undetermined Significance. Sci. World J..

[43] Tanaka Y., Abe M., Hiasa M., Oda A., Amou H., Nakano A., Takeuchi K., Kitazoe K., Kido S., Inoue D. (2007). Myeloma Cell-Osteoclast Interaction Enhances Angiogenesis Together with Bone Resorption: A Role for Vascular Endothelial Cell Growth Factor and Osteopontin. Clin. Cancer Res..

[44] Rajkumar S.V. (2016). Updated Diagnostic Criteria and Staging System for Multiple Myeloma. Am. Soc. Clin. Oncol. Educ. Book.

[45] Greipp P.R., Miguel J.S., Durie B.G.M., Crowley J.J., Barlogie B., Bladé J., Boccadoro M., Child J.A., Avet-Loiseau H., Kyle R.A. (2005). International Staging System for Multiple Myeloma. J. Clin. Oncol..

[46] Gerov V., Gerova D., Micheva I., Nikolova M., Mihaylova G., Galunska B. (2023). Dynamics of Bone Disease Biomarkers Dickkopf-1 and Sclerostin in Patients with Multiple Myeloma. JCM.

[47] Kumar S., Paiva B., Anderson K.C., Durie B., Landgren O., Moreau P., Munshi N., Lonial S., Bladé J., Mateos M.-V. (2016). International Myeloma Working Group Consensus Criteria for Response and Minimal Residual Disease Assessment in Multiple Myeloma. Lancet Oncol..

[48] Rajkumar S.V., Dimopoulos M.A., Palumbo A., Blade J., Merlini G., Mateos M.-V., Kumar S., Hillengass J., Kastritis E., Richardson P. (2014). International Myeloma Working Group Updated Criteria for the Diagnosis of Multiple Myeloma. Lancet Oncol..

[49] Al Saleh A.S., Parmar H.V., Visram A., Muchtar E., Buadi F.K., Go R.S., Dispenzieri A., Kapoor P., Warsame R., Lacy M.Q. (2020). Increased Bone Marrow Plasma-Cell Percentage Predicts Outcomes in Newly Diagnosed Multiple Myeloma Patients. Clin. Lymphoma Myeloma Leuk..

[50] Pearse R.N., Sordillo E.M., Yaccoby S., Wong B.R., Liau D.F., Colman N., Michaeli J., Epstein J., Choi Y. (2001). Multiple Myeloma Disrupts the TRANCE/ Osteoprotegerin Cytokine Axis to Trigger Bone Destruction and Promote Tumor Progression. Proc. Natl. Acad. Sci. USA.

[51] Sordillo E.M., Pearse R.N. (2003). RANK-Fc: A Therapeutic Antagonist for RANK-L in Myeloma. Cancer.

[52] Deleenheer E. (2004). Evidence of a Role for RANKL in the Development of Myeloma Bone Disease. Curr. Opin. Pharmacol..

[53] Schmiedel B.J., Scheible C.A., Nuebling T., Kopp H.-G., Wirths S., Azuma M., Schneider P., Jung G., Grosse-Hovest L., Salih H.R. (2013). RANKL Expression, Function, and Therapeutic Targeting in Multiple Myeloma and Chronic Lymphocytic Leukemia. Cancer Res..

[54] Sfiridaki K., Pappa C.A., Tsirakis G., Kanellou P., Kaparou M., Stratinaki M., Sakellaris G., Kontakis G., Alexandrakis M.G. (2011). Angiogenesis-Related Cytokines, RANKL, and Osteoprotegerin in Multiple Myeloma Patients in Relation to Clinical Features and Response to Treatment. Mediators Inflamm..

[55] Kraj M., Owczarska K., Sokołowska U., Centkowski P., Pogłód R., Kruk B. (2005). Correlation of Osteoprotegerin and sRANKL Concentrations in Serum and Bone Marrow of Multiple Myeloma Patients. Arch. Immunol. Ther. Exp..

[56] Schütt P., Rebmann V., Brandhorst D., Wiefelspütz J., Ebeling P., Opalka B., Seeber S., Nowrousian M.R., Moritz T., Grosse-Wilde H. (2008). The Clinical Significance of Soluble Human Leukocyte Antitgen Class-I, ICTP, and RANKL Molecules in Multiple Myeloma Patients. Hum. Immunol..

[57] Heider U., Langelotz C., Jakob C., Zavrski I., Fleissner C., Eucker J., Possinger K., Hofbauer L.C., Sezer O. (2003). Expression of Receptor Activator of Nuclear Factor kappaB Ligand on Bone Marrow Plasma Cells Correlates with Osteolytic Bone Disease in Patients with Multiple Myeloma. Clin. Cancer Res. Off. J. Am. Assoc. Cancer Res..

[58] Slany A., Haudek-Prinz V., Meshcheryakova A., Bileck A., Lamm W., Zielinski C., Gerner C., Drach J. (2014). Extracellular Matrix Remodeling by Bone Marrow Fibroblast-like Cells Correlates with Disease Progression in Multiple Myeloma. J. Proteome Res..

[59] Lamort A.-S., Giopanou I., Psallidas I., Stathopoulos G.T. (2019). Osteopontin as a Link between Inflammation and Cancer: The Thorax in the Spotlight. Cells.

[60] Dizdar O., Barista I., Kalyoncu U., Karadag O., Hascelik G., Cila A., Pinar A., Celik I., Kars A., Tekuzman G. (2007). Biochemical Markers of Bone Turnover in Diagnosis of Myeloma Bone Disease. Am. J. Hematol..

[61] Valković T., Babarović E., Lučin K., Štifter S., Aralica M., Pećanić S., Seili-Bekafigo I., Duletić-Načinović A., Nemet D., Jonjić N. (2014). Plasma Levels of Osteopontin and Vascular Endothelial Growth Factor in Association with Clinical Features and Parameters of Tumor Burden in Patients with Multiple Myeloma. BioMed Res. Int..

[62] Kang S.Y., Lee J.J., Lee W.I. (2007). Clinical Significance of Serum Osteopontin in Patients with Multiple Myeloma. Ann. Lab. Med..

[63] Maaroufi A., Khadem-Ansari M.-H., Khalkhali H.-R., Rasmi Y. (2020). Serum Levels of Bone Sialoprotein, Osteopontin, and Β2-Microglobulin in Stage I of Multiple Myeloma. J. Cancer Res. Ther..

[64] Sfiridaki A., Miyakis S., Pappa C., Tsirakis G., Alegakis A., Kotsis V., Stathopoulos E., Alexandrakis M. (2011). Circulating Osteopontin: A Dual Marker of Bone Destruction and Angiogenesis in Patients with Multiple Myeloma. J. Hematol. Oncol..

[65] Colla S., Morandi F., Lazzaretti M., Rizzato R., Lunghi P., Bonomini S., Mancini C., Pedrazzoni M., Crugnola M., Rizzoli V. (2005). Human Myeloma Cells Express the Bone Regulating Gene Runx2/Cbfa1 and Produce Osteopontin That Is Involved in Angiogenesis in Multiple Myeloma Patients. Leukemia.

[66] Babarović E., Valković T., Budisavljević I., Balen I., Štifter S., Duletić-Načinović A., Lučin K., Jonjić N. (2016). The Expression of Osteopontin and Vascular Endothelial Growth Factor in Correlation with Angiogenesis in Monoclonal Gammopathy of Undetermined Significance and Multiple Myeloma. Pathol.-Res. Pract..

[67] Robbiani D.F., Colon K., Ely S., Ely S., Chesi M., Bergsagel P.L. (2007). Osteopontin Dysregulation and Lytic Bone Lesions in Multiple Myeloma. Hematol. Oncol..

[68] Liu Z., Zeng Q., Xiang B. (2021). Bortezomib-Based Regimens Improve the Prognosis of Newly Diagnosed MM Patients with Chromosomal Aberrations except for Del(17q13): A Retrospective Study from a Single Center. Medicine.

[69] Eom K.-S., Kim S.J., Lee J.-J., Suh C., Kim J.S., Yoon S.-S., Kim B.S., Kang H.J., Choi Y.J., Kim C.S. (2014). Changes in Osteoblastic Activity in Patient Who Received Bortezomib as Second Line Treatment for Plasma Cell Myeloma: A Prospective Multicenter Study. BioMed Res. Int..

[70] Lin L., Chen D., Xiang Z.-F., Pei R.-Z., Zhang P.-S., Liu X.-H., Du X.-H., Lu Y. (2017). Bortezomib Could Down-Regulate the Expression of RANKL, Inhibit Cell Proliferation and Induce Cell Apoptosis in the Human Myeloma Cell Line RPMI 8226 by Activating Casepase-3. Cancer Biomark..

[71] Terpos E., Heath D.J., Rahemtulla A., Zervas K., Chantry A., Anagnostopoulos A., Pouli A., Katodritou E., Verrou E., Vervessou E.-C. (2006). Bortezomib Reduces Serum Dickkopf-1 and Receptor Activator of Nuclear Factor-? B Ligand Concentrations and Normalises Indices of Bone Remodelling in Patients with Relapsed Multiple Myeloma. Br. J. Haematol..

