# Supplementary material for: Circulating sRANKL, Periostin, and Osteopontin as Biomarkers for the Assessment of Activated Osteoclastogenesis in Myeloma Related Bone Disease
Source: Cancers (Basel). 2023 Nov 24;15(23):5562. doi: 10.3390/cancers15235562 (PMC10705189; doi:10.3390/cancers15235562)
Supplement: Supplementary file 1 [file cancers-15-05562-s001.zip › cancers-2709947-supplementary.pdf]

Supplementary Tables

Table S1. Patients' demographic and clinical characteristics at baseline.

Characteristic	*n* (%)
Patients	41 (100%)
Age (years) *	64.02 ± 12.14
**Gender:**	
Male	20 (48.78%)
Female	21 (51.22%)
**ISS stages:**	
I	16 (39.02%)
II	6 (14.63%)
III	19 (46.34%)
**Bone disease status:**	
G1 (≤3 osteolytic lesions)	9 (21.95%)
G2 (>3 osteolytic lesions + bone fractures)	32 (78.05%)
**MPCs infiltration in bone marrow:**	
<60%	17 (41.46%)
>60%	24 (58.54%)
**M-protein type:**	
IgG	23 (56.10%)
IgA	6 (14.63%)
FLC (κ + λ)	12 (29.27%)
**Routine laboratory parameters**	**Mean ± SD (range)**
Hb (g/L)	100.78 ± 25.36 (55–151)
WBC (×10^9/L)	6.40 ± 2.95 (2.03–14.58)
Plt (×10^9/L)	208.41 ± 103.31 (32–461)
Creatinine (µmol/L)	126.05 ± 91.53 (53–449)
LDH (IU/L)	425.76 ± 370.34 (200–2571)
Total protein (g/L)	92.70 ± 20.99 (55–133)
Albumin (g/L)	36.71 ± 7.58 (19–51)
B2MG (mg/L)	5.62 ± 3.05 (2.0–14.0)
Calcium (mmol/L)	2.42 ± 0.45 (1.84–4.01)

ISS—International Staging System; Ig—immunoglobulin; FLC—free light chain; MPCs—myeloma plasma cells; Hb—hemoglobin; WBC – white blood cells, Plt – platelets; LDH – lactate dehydrogenase; B2MG – ß2 microglobulin (* – age is presented as mean ± SD).

Table S2. Correlations of sRANKL, periostin and osteopontin with some laboratory parameters.

Parameter	B2MG [mg/ml]	BMI [%]	Creatinine [µmol/L]	TP [g/L]	Albumin [g/L]	Hb [g/L]
**sRANKL** [pg/ml]						
Spearman r	**r=0.576**	**r=0.330**	**r=0.353**	**r=0.209**	**r=-0.318**	**r=-0.374**
(95% CI)	(0.317-0.754)	(0.0159-0.585)	(0.041-0.602)	(-0.115-0.493)	(-0.576-0.002)	(-0.617-0.065)
p value	**p<0.0001**	**p=0.0349**	**p=0.0237**	**p=0.1899**	**p=0.0428**	**p=0.0161**
**Periostin** [pg/ml]						
Spearman r	**r=0.779**	**r=0.417**	**r=0.532**	**r=0.458**	**r=-0.654**	**r=-0.486**
(95% CI)	(0.614-0.879)	(0.116-0.648)	(0.2584-0.726)	(0.166-0.676)	(-0.804-0.426)	(-0.695-0.201)
p value	**p<0.0001**	**p=0.0067**	**p<0.0001**	**p=0.0026**	**p<0.0001**	**p=0.0013**
**Osteopontin** [ng/ml]						
Spearman r	**r=0.697**	**r=0.261**	**r=0.605**	**r=0.146**	**r=-0.279**	**r=-0.332**
(95% CI)	(0.488-0.830)	(-0.060-0.533)	(0.357-0.773)	(-0.178-0.442)	(-0.547-0.041)	(-0.586-0.017)
p value	**p<0.0001**	**p=0.0997**	**p<0.0001**	**p=0.3621**	**p=0.0773**	**p=0.0342**

B2MG – ß2 microglobulin, BMI – bone marrow infiltration by MPCs, TP – total protein, Hb – hemoglobin. Spearman correlation analysis was used; statistical significance was indicated at $p < 0.05$.

Table S3. Serum values of sRANKL, Periostin and Osteopontin, measured at different time points.

Parameter	T0 Median (IQR)	T1 Median (IQR)	T2 Median (IQR)	TA Median (IQR)
**sRANKL** [pg/mL]	9.592 (8.033–10.92)	8.575 (7.201–10.180)	9.210 (5.923–10.090)	5.733 (5.441–6.479)
**Periostin** [pg/mL]	648.4 (594.4–809.9)	565.4 (426.9–633.2)	466.2 (305.5–573.2)	407.9 (322.4–447.6)
**Osteopontin** [ng/mL]	596.0 (479.7–793.8)	584.9 (487.9–621.1)	551.0 (383.2–714.1)	346.6 (264.9–387.9)

T0 – at diagnosis; T1 – after 4 cycles of chemotherapy; T2 – after another 4 cycles (a total of 8 cycles) of chemotherapy; TA –3 months after autologous stem cell transplantation.
